# Food Insecurity across the Life-Course and Cognitive Function among Older Mexican Adults

**DOI:** 10.3390/nu14071462

**Published:** 2022-03-31

**Authors:** Joseph L. Saenz, Jamie Kessler, Ehlana Nelson

**Affiliations:** Leonard Davis School of Gerontology, University of Southern California, 3715 McClintock Ave., Los Angeles, CA 90089, USA; jamiekes@usc.edu (J.K.); ennelson@usc.edu (E.N.)

**Keywords:** food insecurity, cognitive function, aging, Mexico, Latin America, MHAS

## Abstract

Background: Food insecurity remains a global public health problem. Experiencing food insecurity is related to poorer cognitive function among older adults. However, few studies have examined how food insecurity, experienced over the life-course, relates to cognitive function among older adults in Mexico. Methods: Data came from the 2015 Mexican Health and Aging Study (*n* = 11,507 adults aged 50 and over). Early- and late-life food insecurity were ascertained by self-report. We evaluated how both measures of food insecurity related to the performance of multiple cognitive tasks (Verbal Learning, Verbal Recall, Visual Scanning, and Verbal Fluency), while controlling for key health and sociodemographic confounders using linear regression. Results: In descriptive analyses, respondents who experienced food insecurity in either early or late life performed significantly worse on all cognitive tasks when compared to the food secure. In models adjusted for health and sociodemographic confounders, early-life food insecurity predicted worse Verbal Learning performance and late-life food insecurity was associated with poorer Visual Scanning performance. Conclusions: Food insecurity was related to poorer cognitive function in a nationally representative sample of older adults in Mexico. However, results suggested that the significance of effects depended on cognitive task and when in the life-course food insecurity was experienced.

## 1. Introduction

Food insecurity exists when there is a lack of access to sufficient, safe, and nutritious food [1]. It affects individuals worldwide and has slowly been on the rise since 2014 [2]. In 2006, the United States Department of Agriculture (USDA) defined food insecurity as a household-level economic and social condition of limited or uncertain access to adequate food [3]. This contrasts with the 2009 food security definition by the United Nations Food and Agriculture Organization (FAO): “when all people, at all times, have physical, social, and economic access to sufficient safe, and nutritious food that meets their dietary needs and food preferences for an active and healthy life” [4]. Food insecurity can differ in terms of severity. Low food security occurs when “households reduce the quality, variety, and desirability of their diets, but the quantity of food intake and normal eating patterns (are) not substantially disrupted” and very low food security involves “eating patterns of one or more household members (which are) disrupted and food intake (is) reduced because the household lack(s) money and other resources for food” [5].

Food insecurity is a key global public health challenge [6]. Approximately 27% of people around the world have been estimated to be food insecure and 11% are severely food insecure. However, food insecurity differs across country-level and individual-level factors. Food insecurity is more common in low- and middle-income versus high-income economies [4] and tends to be highest in Sub-Saharan Africa, Latin America and the Caribbean, and South Asia and lowest in North America, Eastern Europe, and Central Asia [1]. Poverty has a large influence on whether individuals have adequate food access across the globe [4]. Low education, weak social networks, limited social capital, low household income, and unemployment also correlate with one’s likelihood of experiencing food insecurity [1]. In addition, age, the number of children in the household, marital status, and residence in a rural area are associated with a higher probability of people experiencing food insecurity and severe food insecurity around the world [4]. 

Globally, there are overlapping social-demographic factors that lead to food insecurity. However, there are also differences between countries. We focus specifically on food insecurity in Mexico, which is considered an upper-middle income economy. A substantial portion of the Mexican population experiences food insecurity. For example, in 2010, 44.3% of the Mexican population, approximately 49.9 million people, experienced some level of food insecurity. Of that 44.3%, 19.5% of the population experienced very low food insecurity, 14% reported moderate food insecurity, and 10.8% were considered to have severe food insecurity [6]. 

Research in Mexico has reported that the living arrangements, marital status, socioeconomic position, and health status of household members may play a role in the likelihood of household food insecurity. Particularly, having more children, a single/widowed/divorced female head of the household, low education, low income, speaking an indigenous language, and living in a rural community predicted household food insecurity [6]. Socioeconomic resources, particularly education, have been consistently associated with food security because it generally correlates to enhanced socioeconomic position and more financial resources, which may have a beneficial impact on the way resources are managed in the household. 

Food insecurity also has adverse health-related effects. In the United States, children in food insecure households may experience anemia, low nutrient intake, poor overall health, cognitive problems, aggression/behavioral problems, depression, anxiety, and asthma. Older adults experiencing food insecurity may have low nutrient intake, poor health, depression, and more functional limitation [7]. Diabetes and obesity may also be linked to food insecurity. Research in Mexico has reported that diabetes, body mass index (BMI), and waist circumference may increase with food insecurity [8,9]. This may be due to stress-associated increases in cortisol and central adiposity related to food insecurity. People experiencing food insecurity often have fewer economic resources and may not be able to afford foods that adhere to a diabetic diet, so disease severity worsens [7]. Another theory posits that food insecurity is associated with obesity due to the palatable and high calorie foods easily available to populations experiencing food insecurity in addition to a lack of resources and knowledge to change lifestyles or eating patterns [10]

Recent studies have suggested that the negative effects of food insecurity may also extend to cognitive function [11]. These studies have found associations between food insecurity and poorer cognitive function in communities around the world, including Burkina Faso [12], Malaysia [13], China [14], South Africa [15], India [16], Europe [17], and the United States [18,19,20,21]. Furthermore, research has highlighted that food insecurity experienced across the life-course, including early life [12,13,14,17] and in mid and late life [15,16,18,19,20,21] are related to less favorable cognitive health outcomes. Research has also highlighted the importance of considering multiple domains of cognitive function. For instance, a United States study investigating word learning, delayed memory, animal fluency, and digit symbol substitution found that, after adjustment for sociodemographic and health factors, food insecurity was associated with lower cognition on all tasks except delayed memory [20], suggesting that effects of food insecurity may differ by cognitive domain assessed.

Few studies have examined how food insecurity relates to cognitive outcomes in Mexico or Latin America [22], despite a higher prevalence of food insecurity in Latin America [1]. The aim of this analysis is to investigate how food insecurity relates to cognitive function among older adults in Mexico. Many prior studies only consider food insecurity at a single time point. However, this analysis adds to the growing literature on food insecurity and cognition by interrogating how food insecurity in both early and late life may independently relate to late-life cognitive ability. Furthermore, we evaluate food insecurity effects across several cognitive domains, allowing us to develop a more nuanced profile of how life-course food insecurity may impact the cognitive profiles of older adults. Understanding how food insecurity relates to cognitive health among older adults in Mexico is a pressing issue given that the Mexican population aged 65 and over is projected to increase dramatically from 9.5 to 26.4 million from 2019 to 2050 [23], in combination with the considerable number of individuals already experiencing food insecurity in the country [6]. Understanding the specific determinants of food insecurity, not only in Mexico, but also worldwide, can help inform government programs and policies with the goal of increasing household food security and ameliorating hunger.

## 2. Materials and Methods

We used data from the 2015 Mexican Health and Aging Study (MHAS) [24]. The MHAS is a large, nationally representative, household-based study of aging in Mexico, which interviews older adults (age 50+) and their spouses regardless of age. The MHAS has been described in greater detail elsewhere [25]. In the 2015 wave, 14,779 individuals were interviewed. We eliminated 576 who were age ineligible (under age 50) and then eliminated 915 respondents who required a proxy to complete the interview as these respondents did not receive the cognitive tasks we analyze. We then eliminated an additional 1781 respondents who had missing data on independent variables or were missing on all cognitive tasks. This resulted in a final sample size of 11,507.

### 2.1. Food Insecurity

Following prior studies [12], we analyzed the effects of food-insecurity at multiple points in the life-course. First, early-life food insecurity was assessed by asking respondents whether, before age ten, they often went to bed hungry (yes/no). Late-life food insecurity was assessed at the household level by first asking whether, in the past two years, the respondent had enough money to buy food. Those reporting not having enough money to buy food in the past two years were then asked whether the respondent did not eat or ate less because there was not enough food. We consider those reporting not having enough money to buy food and not eating or eating less in the past two years as food insecure. This is consistent with prior work assessing severe food insecurity [26]. Although food insecurity is multidimensional, incorporating access to food, availability of food, utilization, and stability [27], our measure of late-life food insecurity is akin to food access as other dimensions of food insecurity were not assessed in the MHAS. Our measure of early-life food insecurity, based on frequently going to bed hungry, may incorporate multiple dimensions of food insecurity, particularly access, availability, and stability, all of which may have contributed to respondents frequently going to bed hungry before age ten.

### 2.2. Cognitive Function

We analyzed cognitive function across four tasks. First, we considered Verbal Learning, which involved the respondent being read an eight-word list and being asked to recall the words across three trials. The average number of correctly recalled words was calculated, in the range of 0–8, and is included as a measure of immediate memory and executive function. Second, Verbal Recall involved the respondent recalling the eight-word list after a delay, range 0–8. This assesses a respondent’s delayed memory. Third, Visual Scanning involved the respondent identifying a visual stimulus in an array of visual stimuli (range 0–60). The Visual Scanning task is intended to assess a respondent’s attention and executive function [28]. Last, Verbal Fluency was assessed by having respondents list as many animals as they could in a one-minute time frame (range 0–60). This was included as a measure of language and executive function. Importantly, these measures were selected for study because they do not involve mathematical skills or literacy [29], which is integral when studying cognition in settings with low levels of formal education. 

### 2.3. Covariates

An individual’s likelihood of experiencing food insecurity is dependent on several factors, including demographics, socioeconomic status (SES), living arrangements, rural/urban dwelling, and health status [4,6]. These factors may confound potential associations between food insecurity and cognitive function given their associations with cognitive function [30,31,32,33]. Thus, we adjusted for several potential confounders. 

Focusing first on SES, we adjusted for years of education, income decile, and household wealth decile. Additionally, several yes/no items were used to assess early-life SES, including whether, before age ten, respondents: had an in-home toilet (reverse coded), wore shoes regularly (reverse coded), had siblings drop out of school to assist parents, had family members sleep in the kitchen, and received economic assistance due to financial problems. These items were summed to construct a measure of low early-life SES, with a range of 0–5, with higher values indicating lower early-life SES [34].

Several variables were included to assess living arrangements and rural/urban dwelling. We included the number of residents living in the home and marital status (married/partnered, widowed, or divorced/separated/never married) to capture living arrangements. Rural/urban dwelling was measured by the number of inhabitants in a respondent’s community of residence and categorized as 100,000+ residents, 15,000–99,999 residents, 2500–14,999 residents, or <2500 residents.

Health status was captured using a count of chronic conditions (diabetes, hypertension, stroke, heart attack, respiratory illness, and cancer). BMI was calculated using self-reported height and weight and was classified as underweight (BMI: <18.5), normal (BMI: 18.5–24.9), overweight (BMI: 25–29.9), and obese (BMI: 30+). An additional category was created for individuals with a missing BMI. Self-report of height and weight has been validated in this sample [35].

Lacking social capital [4] is related to a higher likelihood of experiencing food insecurity. Thus, we assessed social capital as whether a respondent reported being able to rely on friends or neighbors for their daily activities (yes/no). In Mexico, food insecurity is also more common among those who speak an indigenous dialect [6]. We further adjusted for whether an individual reported speaking an indigenous dialect. Basic demographic covariates included age and gender. 

### 2.4. Statistical Approach

We first conducted two sets of descriptive analyses by comparing the characteristics of individuals who did versus did not experience food insecurity in early life, and then comparing those who did versus did not experience food insecurity in late life. We then used ordinary least squares regression (OLS) to determine how early- and late-life food insecurity related to cognitive function after adjusting for the covariates outlined above. For each cognitive outcome (Verbal Learning, Verbal Recall, Visual Scanning, and Verbal Fluency) we estimated three models. First, cognitive outcomes were regressed on early-life food insecurity and all covariates to obtain the effects of early-life food insecurity after adjustment for potential confounders. Second, cognitive outcomes were regressed on late-life food insecurity and all covariates to obtain effects of late-life food insecurity after adjustment for potential confounders. Third, cognitive outcomes were regressed on early- and late-life food insecurity and all potential confounders to determine whether food insecurity in early and late life convey independent effects on cognitive function above and beyond each other. Models were estimated using Stata 17 MP.

## 3. Results

### 3.1. Descriptive Results

We present our descriptive results, stratified by those who were food secure versus those who were food insecure by both early- and late-life food insecurity in Table 1. Approximately 29.7% of the sample reported food insecurity in early life, whereas 14.1% reported food insecurity in late-life. Across all cognitive tasks, those who experienced food insecurity in early and late life had significantly lower test scores than those who did not experience food insecurity in early and late life, respectively. Further, individuals who experienced food insecurity had fewer years of education whether one considered early-life (6.7 vs. 3.9) or late-life (6.2 vs. 4.1) food insecurity. Individuals who reported food insecurity, whether in early or late life, were more likely to live in rural areas at the time of the survey. Respondents who were food insecure also appeared more socioeconomically disadvantaged, having lower early-life SES and lower current levels of income and wealth at the time of the survey compared to those who were food secure.

### 3.2. Regression Results

Results from regression analyses for each cognitive task are shown in Table 2. Focusing first on Verbal Learning, in Model 1, early-life food insecurity and all covariates are included. Experiencing food insecurity in early life was significantly associated with lower scores on the Verbal Learning task (*β* = −0.05, *p* < 0.05) even with adjustment for all covariates. However, late-life food insecurity was not significantly related with Verbal Learning performance in Model 2 (*β* = −0.01, *p* > 0.05). Similarly, in Model 3, when both early- and late-life food insecurity were included with all covariates, only early-life food insecurity was significantly associated with Verbal Learning performance (*β* = −0.05, *p* < 0.05), suggesting an independent effect of early-life food insecurity above and beyond late-life food insecurity and all covariates.

We now shift our attention to models with Verbal Recall as an outcome (Models 4–6). In Models 4 and 5, when food insecurity measures were included individually with adjustment for covariates, neither early-life (*β* = −0.04, *p* > 0.05) nor late-life (*β* = −0.03, *p* > 0.05) food insecurity were related to Verbal Recall performance. Furthermore, parameter estimates for early- and late-life food insecurity were not statistically significant in Model 6 when both measures of food insecurity were included simultaneously.

Regression models with Visual Scanning scores as dependent variables are provided in Models 7–9. Model 7 indicated no significant association between early-life food insecurity and Visual Scanning performance (*β* = −0.02, *p* > 0.05) when only early-life food insecurity and covariates were included. However, Model 8, in which only late-life food insecurity and covariates were included, showed that experiencing food insecurity in late-life was related to worse performance on the Visual Scanning task (*β* = −0.76, *p* < 0.05). In Model 9, when both food insecurity measures were included along with covariates, late-life food insecurity remained a significant predictor of worse Visual Scanning performance.

We now focus on regression results for Verbal Fluency in Models 10–12. Results regarding Verbal Fluency did not find support for associations between food insecurity and Verbal Fluency. In Models 10 and 11, neither early-life (*β* = −0.02, *p* > 0.05) nor late-life food insecurity (*β* = 0.05, *p* > 0.05) were associated with Verbal Fluency performance when adjusting for covariates. Model 12, which included both food insecurity measures and covariates, similarly showed that neither measure of food insecurity was related with Verbal Fluency.

## 4. Discussion

Food insecurity remains a significant public health challenge [6], with potential negative effects on health [7,8,9]. Recent work has provided evidence that the health effects of food insecurity may extend to the brain and cognitive function [11,36]. Our analyses add to the growing body of literature on food insecurity and cognition by suggesting that the association may be complex and may depend on cognitive domain and the stage of the life-course in which food insecurity is experienced. Furthermore, recent reviews of literature on food insecurity and cognition underscore the shortage of research focusing on populations in Mexico or Latin America in general [11,36]. Our work extends existing research by exploring associations in the context of Mexico. We found that early-life food insecurity predicted poorer Verbal Learning performance whereas late-life food insecurity related to scoring lower on the Visual Scanning task, even when controlling for relevant socio-demographic, SES, and health confounders. 

Although the mechanisms through which food insecurity may ultimately impact cognitive ability remain an open research question, several have been hypothesized. Considering early-life food insecurity, childhood represents a vital stage in brain development, and experiencing malnutrition may impair the development of the brain, leading to long-lasting impacts on an individual’s brain and cognition [37]. Early-life food insecurity may additionally place individuals at risk of developing chronic conditions, including diabetes and hypertension, later in life [38,39,40], which may ultimately impact the brain and cognitive function [30,32]. Food insecurity, whether experienced in early or late life, also represents a significant stressor [41]. Short-term stress may be beneficial for cognitive function; however, chronic stress may have deleterious effects on cognition [42]. This may occur through elevation in cortisol associated with stress with subsequent effects on brain structures, including the hippocampus [43]. Food insecurity is also related to lower diet quality and nutrient intake [44], including in Mexican samples [45,46], as individuals may respond to limited food access by opting for foods that are cheaper, but not necessarily nutritious. High quality diets have been associated with better cognitive outcomes, including lower risk of Alzheimer’s Disease [47]. This implies that stress and poor diet quality may be potential factors connecting food insecurity to poor cognitive outcomes.

We found that early-life food insecurity was related to poorer Verbal Learning performance, but not with Verbal Recall, Visual Scanning, or Verbal Fluency in our regression analyses. Although many studies of early-life food insecurity have focused on dementia or cognitive impairment rather than cognitive domains [12,13,14], our results differ somewhat from those reported in prior work [17]. Though our finding of early-life food insecurity impacts on Verbal Learning are consistent, Cohn-Schwartz & Weinstein (2020) also found impacts on Verbal Recall and Verbal Fluency performance, even when adjusting for relevant health and socioeconomic confounders. However, early-life hunger in the Cohn-Schwartz & Weinstein (2020) study involved calculating specific ages at which hunger occurred (age 0–4, 5–11, 12–18, or 19+) making it difficult to compare findings with our analyses, which only referred to usually going to bed hungry before age ten. Cohn-Schwartz & Weinstein (2020) also highlight that hunger duration was negatively related to cognitive function. Our binary measure of early-life hunger may not capture important information on duration of hunger, which may weaken our ability to detect the effects on cognitive function.

Regarding late-life food insecurity, our regression analyses suggested that late-life food insecurity was related to worse Visual Scanning performance, whereas associations with other cognitive tasks were not statistically significant. Visual Scanning involves multiple cognitive processes including attention and executive function [28], and several studies have demonstrated that executive function may be a cognitive domain that is particularly vulnerable to the effects of food insecurity [19,20,21]. Furthermore, late-life food insecurity was not related to the performance of memory tasks in our analyses (Verbal Learning or Verbal Recall). This is consistent with prior work finding null associations between late-life food insecurity and memory [19,21]. Wong et al. (2016), who similarly found larger impacts of food insecurity on executive function than memory, hypothesized that this may be because the prefrontal cortex, which is integral for executive function, may be especially affected by stress [48], explaining the effect of stress on executive function [49].

This study has limitations. First, the MHAS survey did not include measures of stress so we could not incorporate stress in our analyses. Food insecurity may be a significant stressor that affects both physical and mental health. Future studies should examine whether the effects of food insecurity on cognitive function may operate through stress. Second, a direct assessment of diet and nutrient intake of the participants was also not available. Future studies on this topic should record the typical food intake of the participants to analyze if the quality of food and nutrient intake may explain the negative correlation between food insecurity and cognition. Measures of food insecurity should also be broadened to include the assessments of specific ages and durations of food insecurity. Third, evaluation of participants’ cognitive function relied on cognitive test performance because the MHAS does not conduct neuroimaging of respondents. Having access to imaging data through magnetic resonance imaging may give insight into the direct effects of food insecurity on the brain, including specific regions, to better understand the potential mechanisms of food insecurity effects. Fourth, although models controlled for economic, demographic, and social characteristics, some confounding variables may still exist. Last, our analyses were based on 2015 data, before the COVID-19 pandemic increased food insecurity in Mexico [50]. Future studies should investigate the effects of food insecurity on cognition during the COVID-19 pandemic. Notwithstanding these limitations, our analysis comes with strengths, including the use of a large nationally representative sample of older adults, assessment of food insecurity throughout the life-course, cognitive data encompassing multiple cognitive domains, and our ability to adjust for a wide range of sociodemographic and health covariates in our models.

## 5. Conclusions

Our findings on the association between food insecurity and cognition have implications for public policy. It is necessary to determine interventions that target food insecurity with the goal of increasing household food security and improving overall health and cognition. One study of a Mexican sample aged 70 and over explored the impacts of supplemental income on cognition and found improvements in immediate and delayed memory, which were mediated through food availability [22]. Supplemental income programs typically target reducing poverty, but may also improve healthcare use, food availability, and decrease anemia. This suggests supplemental income programs may be an effective strategy to improve cognitive function by reducing known risk factors for cognitive impairment such as cardiovascular disease, diabetes, hypertension, depression, malnutrition, and anemia. Studies in the United States have similarly found program interventions may reduce food insecurity. Positive impacts of new Supplemental Nutrition Assistance Program (SNAP) enrollment have been found on dietary outcomes and food insecurity among older adult Supplemental Security Income (SSI) recipients with participation in SNAP and SSI, improving food insecurity and accessibility, allowing participants to choose more nutritious options, freeing up budgets to spend money on other non-food related necessities, and easing mental distress [51]. Income benefits may then impact cognition through food security, consumption of nutrients, and reduction of stress. Programs should be designed to target food insecurity in early and late life, particularly in low- and middle-income countries where food insecurity is particularly high. 

## Figures and Tables

**Table 1:** Sociodemographic, cognitive, and health characteristics of Mexican adults (age 50+) by food insecurity.

	Early Life					Late Life				
	Food Secure (*n* = 8084)		Food Insecure (*n* = 3423)			Food Secure (*n* = 9890)		Food Insecure (*n* = 1617)		
	Mean	SD	Mean	SD	Sig	Mean	SD	Mean	SD	Sig
**Cognitive Function**										
Verbal Learning	4.9	1.2	4.5	1.2	***	4.8	1.2	4.6	1.2	***
Verbal Recall	4.4	2.1	3.8	2.2	***	4.2	2.1	4.0	2.2	***
Visual Scanning	31.1	15.8	24.6	14.7	***	29.9	15.8	25.1	15.0	***
Verbal Fluency	16.0	5.3	14.5	4.9	***	15.7	5.3	14.6	5.0	***
**Demographics**										
Age	65.8	9.5	67.2	9.5	***	66.4	9.5	65.3	9.3	***
Years of Education	6.7	4.9	3.9	3.6	***	6.2	4.8	4.1	3.6	***
Female (*n*, %)	4863	60.2	1903	55.6	***	5783	58.5	983	60.8	
Persons in Household	2.4	1.7	2.6	1.9	***	2.4	1.7	2.7	2.0	***
Low Early Life SES	1.3	1.1	2.5	1.1	***	1.6	1.2	2.1	1.2	***
Speaks Indigenous Dialect (*n*, %)	429	5.3	377	11.0	***	635	6.4	171	10.6	***
Income Decile	4.6	3.1	4.1	2.7	***	4.6	3.0	3.5	2.6	***
Wealth Decile	4.7	2.9	4.1	2.8	***	4.7	2.8	3.6	2.8	***
**Locality Size**										
100,000+ (*n*, %)	5152	63.7	1592	46.5	***	5986	60.5	758	46.9	***
15,000–99,999 (*n*, %)	1024	12.7	478	14.0		1291	13.1	211	13.0	
2500–14,999 (*n*, %)	679	8.4	408	11.9		883	8.9	204	12.6	
<2500 (*n*, %)	1229	15.2	945	27.6		1730	17.5	444	27.5	
**Marital Status**										
Married/Partnered (*n*, %)	5377	66.5	2248	65.7	*	6605	66.8	1020	63.1	**
Widowed (*n*, %)	1491	18.4	699	20.4		1860	18.8	330	20.4	
Other (Divorced/Separated/Never) (*n*, %)	1216	15.0	476	13.9		1425	14.4	267	16.5	
**Social Capital**										
Can rely on friends/neighbors (*n*, %)	4828	59.7	1938	56.6	**	5897	59.6	869	53.7	***
**Health Status**										
Chronic Condition Count	1.1	1.0	1.2	1.0	***	1.1	1.0	1.2	1.0	***
**Body Mass Index**										
Underweight (*n*, %)	86	1.1	53	1.5	***	107	1.1	32	2.0	***
Normal (*n*, %)	2095	25.9	913	26.7		2600	26.3	408	25.2	
Overweight (*n*, %)	3047	37.7	1153	33.7		3672	37.1	528	32.7	
Obese (*n*, %)	1966	24.3	750	21.9		2360	23.9	356	22.0	
Missing (*n*, %)	890	11.0	554	16.2		1151	11.6	293	18.1	

Note: Authors’ own calculation using data from the 2015 Mexican Health and Aging Study. SD: standard deviation. SES: socioeconomic status. Sig. represents whether differences in variables by experience of food insecurity were statistically significant at * *p* < 0.05, ** *p* < 0.01, *** *p* < 0.001.

**Table 2:** Ordinary Least Squares Regressions of Cognitive Domains among Older Mexican Adults.

	Verbal Learning			Verbal Recall			Visual Scanning			Verbal Fluency		
	Model 1	Model 2	Model 3	Model 4	Model 5	Model 6	Model 7	Model 8	Model 9	Model 10	Model 11	Model 12
**Food Insecurity**	*β* (SE)	*β* (SE)	*β* (SE)	*β* (SE)	*β* (SE)	*β* (SE)	*β* (SE)	*β* (SE)	*β* (SE)	*β* (SE)	*β* (SE)	*β* (SE)
Early-Life	−0.05 *		−0.05 *	−0.04		−0.04	−0.02		0.01	−0.02		−0.02
	(0.02)		(0.02)	(0.04)		(0.04)	(0.28)		(0.28)	(0.10)		(0.10)
Late-Life		−0.01	−0.00		−0.03	−0.03		−0.76 *	−0.76 *		0.05	0.05
		(0.03)	(0.03)		(0.05)	(0.05)		(0.33)	(0.33)		(0.12)	(0.12)
**Covariates**												
Age	−0.04 ***	−0.04 ***	−0.04 ***	−0.07 ***	−0.07 ***	−0.07 ***	−0.54 ***	−0.55 ***	−0.55 ***	−0.12 ***	−0.12 ***	−0.12 ***
	(0.00)	(0.00)	(0.00)	(0.00)	(0.00)	(0.00)	(0.01)	(0.01)	(0.01)	(0.01)	(0.01)	(0.01)
Female	0.37 ***	0.37 ***	0.37 ***	0.69 ***	0.69 ***	0.69 ***	0.21	0.19	0.19	−0.26 **	−0.26 **	−0.26 **
	(0.02)	(0.02)	(0.02)	(0.04)	(0.04)	(0.04)	(0.25)	(0.25)	(0.25)	(0.09)	(0.09)	(0.09)
Years of Education	0.07 ***	0.07 ***	0.07 ***	0.09 ***	0.09 ***	0.09 ***	1.30 ***	1.30 ***	1.30 ***	0.34 ***	0.34 ***	0.34 ***
	(0.00)	(0.00)	(0.00)	(0.00)	(0.00)	(0.00)	(0.03)	(0.03)	(0.03)	(0.01)	(0.01)	(0.01)
Locality Size 15,000–99,999 (Ref: 100,000+)	−0.02	−0.02	−0.02	−0.02	−0.02	−0.02	−1.25 ***	−1.25 ***	−1.25 ***	−0.13	−0.13	−0.13
	(0.03)	(0.03)	(0.03)	(0.05)	(0.05)	(0.05)	(0.34)	(0.34)	(0.34)	(0.13)	(0.13)	(0.13)
Locality Size 2500–14,999 (Ref: 100,000+)	−0.11 **	−0.11 **	−0.11 **	−0.03	−0.03	−0.03	−3.44 ***	−3.41 ***	−3.41 ***	−0.60 ***	−0.60 ***	−0.60 ***
	(0.03)	(0.03)	(0.03)	(0.06)	(0.06)	(0.06)	(0.39)	(0.39)	(0.39)	(0.15)	(0.15)	(0.15)
Locality Size < 2500 (Ref: 100,000+)	−0.18 ***	−0.18 ***	−0.18 ***	−0.18 ***	−0.18 ***	−0.18 ***	−4.23 ***	−4.21 ***	−4.21 ***	−0.58 ***	−0.59 ***	−0.58 ***
	(0.03)	(0.03)	(0.03)	(0.05)	(0.05)	(0.05)	(0.32)	(0.32)	(0.32)	(0.12)	(0.12)	(0.12)
Widowed (Ref: Married/Partnered)	−0.04	−0.04	−0.04	0.02	0.02	0.02	−1.33 ***	−1.31 ***	−1.31 ***	−0.18	−0.18	−0.18
	(0.03)	(0.03)	(0.03)	(0.05)	(0.05)	(0.05)	(0.33)	(0.33)	(0.33)	(0.12)	(0.12)	(0.12)
Div/Sep/Never (Ref: Married/Partnered)	−0.05	−0.05	−0.05	−0.06	−0.06	−0.05	−0.98 **	−0.95 **	−0.95 **	−0.38 **	−0.38 **	−0.38 **
	(0.03)	(0.03)	(0.03)	(0.05)	(0.05)	(0.05)	(0.33)	(0.33)	(0.33)	(0.12)	(0.12)	(0.13)
Can Rely on Others	0.10 ***	0.10 ***	0.10 ***	0.17 ***	0.17 ***	0.17 ***	0.55 *	0.54 *	0.54 *	0.45 ***	0.46 ***	0.46 ***
	(0.02)	(0.02)	(0.02)	(0.04)	(0.04)	(0.04)	(0.22)	(0.22)	(0.22)	(0.08)	(0.08)	(0.08)
Residents in Home	−0.01 *	−0.01 *	−0.01 *	0.01	0.01	0.01	−0.35 ***	−0.34 ***	−0.34 ***	−0.06 *	−0.06 *	−0.06 *
	(0.01)	(0.01)	(0.01)	(0.01)	(0.01)	(0.01)	(0.06)	(0.06)	(0.06)	(0.02)	(0.02)	(0.02)
Low Early Life SES	−0.02 *	−0.03 ***	−0.02 *	−0.04 *	−0.05 **	−0.04 *	−0.44 ***	−0.43 ***	−0.43 ***	0.01	0.01	0.01
	(0.01)	(0.01)	(0.01)	(0.02)	(0.02)	(0.02)	(0.11)	(0.10)	(0.11)	(0.04)	(0.04)	(0.04)
Speaks Indigenous Dialect	−0.19 ***	−0.19 ***	−0.19 ***	−0.05	−0.05	−0.05	−3.06 ***	−3.05 ***	−3.05 ***	−1.44 ***	−1.44 ***	−1.44 ***
	(0.04)	(0.04)	(0.04)	(0.07)	(0.07)	(0.07)	(0.45)	(0.45)	(0.45)	(0.17)	(0.17)	(0.17)
Income Decile	0.02 ***	0.02 ***	0.02 ***	0.01 *	0.01 *	0.01 *	0.27 ***	0.26 ***	0.26 ***	0.09 ***	0.09 ***	0.09 ***
	(0.00)	(0.00)	(0.00)	(0.01)	(0.01)	(0.01)	(0.04)	(0.04)	(0.04)	(0.02)	(0.02)	(0.02)
Wealth Decile	0.01 *	0.01 *	0.01 *	0.00	0.00	0.00	0.24 ***	0.23 ***	0.23 ***	0.05 ***	0.05 ***	0.05 ***
	(0.00)	(0.00)	(0.00)	(0.01)	(0.01)	(0.01)	(0.04)	(0.04)	(0.04)	(0.02)	(0.02)	(0.02)
Chronic Condition Count	−0.02	−0.02 *	−0.02	−0.06 **	−0.06 **	−0.06 **	−0.96 ***	−0.95 ***	−0.95 ***	−0.22 ***	−0.22 ***	−0.22 ***
	(0.01)	(0.01)	(0.01)	(0.02)	(0.02)	(0.02)	(0.12)	(0.12)	(0.12)	(0.04)	(0.04)	(0.04)
BMI: Underweight (Ref: Normal)	−0.15	−0.16	−0.15	−0.33 *	−0.33 *	−0.33 *	−3.58 **	−3.52 **	−3.52 **	−1.15 **	−1.16 **	−1.15 **
	(0.09)	(0.09)	(0.09)	(0.16)	(0.16)	(0.16)	(1.12)	(1.12)	(1.12)	(0.39)	(0.39)	(0.39)
BMI: Overweight (Ref: Normal)	0.09 ***	0.09 ***	0.09 ***	0.09 *	0.09 *	0.09 *	1.09 ***	1.09 ***	1.09 ***	0.29 **	0.29 **	0.29 **
	(0.02)	(0.02)	(0.02)	(0.05)	(0.05)	(0.05)	(0.28)	(0.28)	(0.28)	(0.11)	(0.11)	(0.11)
BMI: Obese (Ref: Normal)	0.14 ***	0.14 ***	0.14 ***	0.11 *	0.11 *	0.11 *	1.40 ***	1.39 ***	1.39 ***	0.57 ***	0.57 ***	0.57 ***
	(0.03)	(0.03)	(0.03)	(0.05)	(0.05)	(0.05)	(0.32)	(0.32)	(0.32)	(0.12)	(0.12)	(0.12)
BMI: Missing (Ref: Normal)	−0.20 ***	−0.20 ***	−0.20 ***	−0.50 ***	−0.50 ***	−0.50 ***	−2.88 ***	−2.86 ***	−2.86 ***	−0.99 ***	−1.00 ***	−1.00 ***
	(0.03)	(0.03)	(0.03)	(0.06)	(0.06)	(0.06)	(0.40)	(0.40)	(0.40)	(0.15)	(0.15)	(0.15)
Observations	11,474	11,474	11,474	11,453	11,453	11,453	10,765	10,765	10,765	11,479	11,479	11,479

Note. Authors’ own calculation using data from the 2015 Mexican Health and Aging Study. *β* indicates unstandardized parameter estimate. * indicates *p* < 0.05, ** indicates *p* < 0.01, *** indicates *p* < 0.001. BMI: body mass index.

## Data Availability

The data files and documentation of data used in this study are public use and available at www.MHASweb.org. Accessed 25 March 2022.
